# Evaluation of the relationship between previous statin use and thyroid cancer using Korean National Health Insurance Service-Health Screening Cohort data

**DOI:** 10.1038/s41598-021-87297-6

**Published:** 2021-04-12

**Authors:** So Young Kim, Young Shin Song, Jee Hye Wee, Chanyang Min, Dae Myoung Yoo, Chang-Ho Lee, Chang Myeon Song, Bumjung Park, Hyo Geun Choi

**Affiliations:** 1 Department of Otorhinolaryngology-Head & Neck Surgery, CHA Bundang Medical Center, CHA University, Seongnam, Korea; 2 Department of Internal Medicine, CHA Bundang Medical Center, CHA University, Seongnam, Korea; 3 Department of Otorhinolaryngology-Head & Neck Surgery, Hallym University College of Medicine, Anyang, Korea; 4 Hallym Data Science Laboratory, Hallym University College of Medicine, Anyang, Korea; 5 Graduate School of Public Health, Seoul National University, Seoul, Korea; 6 Department of Otorhinolaryngology-Head & Neck Surgery, Hanyang University College of Medicine, Seoul, Korea

**Keywords:** Cancer, Oncology

## Abstract

The association of thyroid cancer with statin use is controversial. This study aimed to investigate the association of previous statin use with thyroid cancer in the ≥ 40-year-old population in the Korean National Health Insurance Service-Health Screening Cohort. The 5501 patients in the thyroid cancer group were matched with the 22,004 patients in the non-thyroid cancer group for age, sex, income, and region of residence. Previous statin use during the 2 years before the diagnosis of thyroid cancer was examined. The odds ratios (ORs) with 95% confidence intervals (CIs) of previous statin use for thyroid cancer were estimated using conditional logistic regression analyses. Additionally, subgroup analyses were conducted. The thyroid cancer group showed more days of previous statin use than the non-thyroid cancer group (72.3, standard deviation [SD] = 181.2 days vs. 64.3, SD = 174.4 days, P = 0.003). Although the odds of previous statin use for thyroid cancer were high in the crude model (OR = 1.10, 95% CI 1.04–1.17, P = 0.002), they were low in the fully adjusted model (OR = 0.89, 95% CI 0.82–0.95, P = 0.001). According to age and sex subgroups, the younger (< 60 years old) male group showed lower odds for thyroid cancer according to previous statin use (adjusted OR = 0.70, 95% CI 0.55–0.88, P = 0.003), but this finding was not observed in other subgroups of older men or in any groups of women. Thyroid cancer was negatively associated with statin use in the previous 2 years in the adjusted model.

## Introduction

Statins are lipid-lowering agents that act as a 3-hydroxy-3-methylglutaryl coenzyme A (HMG-CoA) reductase^1^. In addition to being therapeutic agents for dyslipidemia, a number of studies have reported their pleotropic effects, including anti-inflammatory^2^, immune-modulating^3^, and anticancer effects^4–6^. Many previous studies have suggested that statins have anticancer effects in colorectal cancer^7^, breast cancer^8^, endocrine-related gynecological cancer, and ovarian cancer, with some conflicting results^9^.

Thyroid cancer is one of the most prevalent cancers worldwide. The incidence of thyroid cancer has been estimated to be approximately 14.42 (95% confidence interval [95% CI] 14.20–14.64) per 100,000 person-years in the US from 2010 to 2013^10^. In Korea, the incidence of total thyroidectomy increased from 2.6 (95% CI 2.5–2.8) per 100,000 person-years in 2007 to 70.1 (95% CI 69.4–70.9) per 100,000 person-years in 2012 and then decreased to 23.6 (95% CI 23.2–24.0) per 100,000 person-years in 2016^11^. Multiple risk factors have been reported, such as female sex, family history of thyroid cancer, and obesity, while the effects of smoking and alcohol consumption on thyroid cancer have been controversial^12^. In particular, it was supposed that there is a sex difference in thyroid cancer due to the effects of estrogen on the thyroid gland^13,14^.

The impacts of statins on thyroid cancer have been controversial^15^. Several in vitro or in vivo studies have demonstrated protective effects of statins against thyroid cancer^16–18^. As with other types of tumors, tumor cell cycle arrest at the G1 phase and apoptosis promotion have been suggested as possible molecular mechanisms for the anticancer effects of statins in thyroid cancer^16–18^. Interestingly, cell cycle arrest by statins was prevented by estrogen in breast cancer cell lines^19,20^. Thus, the different anticancer effects of statins could be anticipated according to sex. On the other hand, a previous case–control study in Taiwan demonstrated that statin medication was linked with an increased rate of thyroid cancer^21^. However, the authors did not adjust for possible confounders such as past medical histories and the life-habit factors of body mass index (BMI), tobacco smoking, and liquor drinking^21^.

If previous stain use might be able to contribute to decreasing the high prevalence of thyroid cancer, the prescription of statins could be a way to prevent thyroid cancer. Thus, we hypothesized that previous statin use could have beneficial effects on the occurrence of thyroid cancer. To minimize potential confounding effects of past medical histories, obesity, smoking habit, and alcohol drinking, these variables were adjusted in the analysis of the relationship between previous statin use and thyroid cancer. In addition, because of the sex differences in thyroid cancer, subgroup analyses were implemented in accordance with age and sex, and other variables were specified for the subgroup analyses.

## Materials and methods

### Ethics

This study was approved by the ethics committee of Hallym University (2019-10-023). The obligation for written informed consent was relinquished by the IRB. All analyses obeyed the instructions and guidelines of the IRB. All methods were carried out in accordance with relevant guidelines and regulations.

### Study population and participant selection

**Figure 1:**
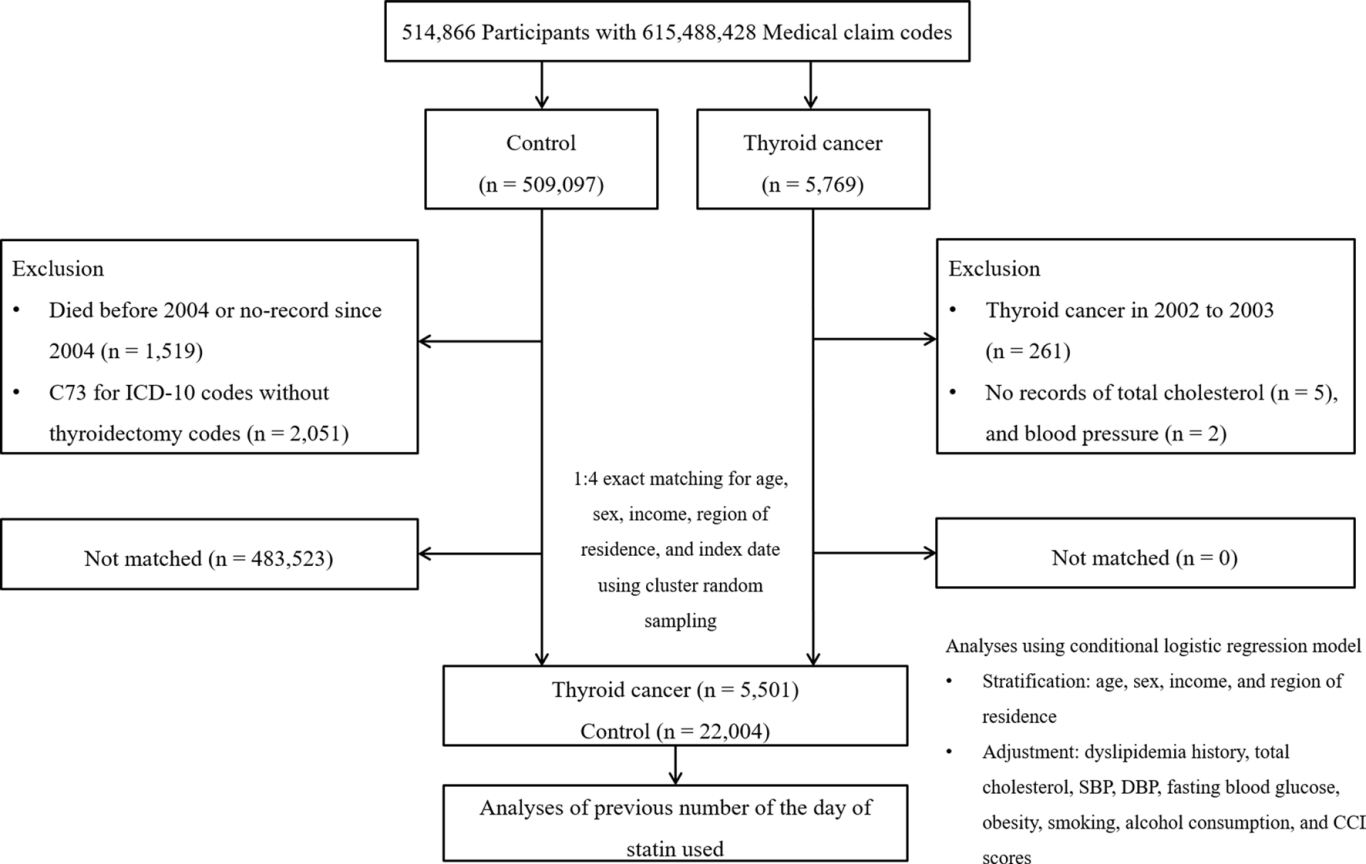
A schematic illustration of the participant selection process that was used in the present study. Of a total of 514,866 participants, 5501 thyroid cancer participants were matched with 22,004 non-thyroid cancer participants for age, sex, income, and region of residence. *DBP* diastolic blood pressure, *SBP* systolic blood pressure. Thyroid cancer: the thyroid cancer group comprised participants assigned the C73 ICD-10 code who underwent thyroidectomy.

A descriptive explanation of the Korean National Health Insurance Service-Health Screening Cohort data is provided elsewhere^22^. Thyroid cancer participants were chosen from 514,866 participants with 615,488,428 medical claim codes (n = 5769). The non-thyroid cancer group was composed of the participants who were not thyroid cancer patients (n = 509,097). To measure statin prescription dates over a period of 2 years, we excluded participants with thyroid cancer who were diagnosed with thyroid cancer between 2002 and 2003 (n = 261). Among the thyroid cancer participants, we excluded those without records of total cholesterol (n = 5) and blood pressure (n = 2). Among the non-thyroid cancer participants, we excluded those who died before 2004 or had no records since 2004 (n = 1519) and those who were treated for thyroid cancer (ICD-10 codes: C73) without thyroidectomy (n = 2051). Thyroid cancer participants were 1:4 matched with non-thyroid cancer participants for age, sex, income, and region of residence. To diminish selection bias, the non-thyroid cancer participants were selected with random sampling. We selected matched control participants in a random order according to age, sex, income, and region of residence. The index date of each thyroid cancer participant was set as the time of diagnosis of thyroid cancer. The index date of non-thyroid cancer participants was set as the index date of the matched thyroid cancer participants. During the matching process, 483,523 non-thyroid cancer participants were excluded. Finally, 5501 thyroid cancer participants were 1:4 matched with 22,004 non-thyroid cancer participants (Fig. 1).

### Exposure (dates of statin prescription)

The sum of the total dates of statin prescription 2 years (730 days) before the index dates was considered the continuous variable. Atorvastatin, pitavastatin, pravastatin, rosuvastatin, fluvastatin, lovastatin, and simvastatin were included. Pravastatin and rosuvastatin were categorized as hydrophilic statins, and atorvastatin, fluvastatin, lovastatin, pitavastatin, and simvastatin were categorized as lipophilic statins.

### Outcome (thyroid cancer)

Participants were considered to have thyroid cancer if they were diagnosed with thyroid cancer (ICD-10 codes: C73). Among the patients, we chose only the participants treated with thyroidectomy (claim codes: P4551 [Total Thyroidectomy-Unilateral], P4552 [Total Thyroidectomy-Bilateral], P4553 [Subtotal Thyroidectomy-Unilateral], P4554 [Subtotal Thyroidectomy-Bilateral], and P4561 [Radical Operation of Malignant Thyroid Tumor]) following our previous studies^23,24^.

### Covariates

Age was categorized into 5-year intervals from 40 to 44 through 85+ years old. Income groups were divided into five classes from lowest income to highest income. The region of residence was grouped into urban and rural areas following our previous study^25^. Cigarette smoking, alcohol drinking, and obesity, classified in line with body mass index (BMI, kg/m^2^), were categorized in the same way as in our previous study^26^. Systolic/diastolic blood pressure (SBP and DBP, respectively), total cholesterol, and fasting blood glucose were measured within a year before the index date. Dyslipidemia was defined according to the recording of ICD-10 code E78 ≥ 2 times before the index date.

The Charlson Comorbidity Index (CCI) was used to measure 17 categories of disease entity except thyroid cancer and was analyzed as a continuous variable (0 [no disease] through 29 [multiple diseases])^27^.

### Statistical analyses

The general characteristics were compared between thyroid cancer and non-thyroid cancer. The chi-square test was used for categorical variables, and the independent *t* test was used for continuous variables.

To estimate the odds ratios (ORs) with 95% CIs of the sum of days of statin prescription (in 1 year) for thyroid cancer, conditional logistic regression was used. In this analysis, crude, model 1 (adjusted for SBP, DBP, dyslipidemia history, total cholesterol and fasting blood glucose) and model 2 (adjusted for model 1 plus CCI scores, smoking, alcohol consumption, and obesity) were calculated. The analysis was stratified by age, sex, income (lower income [1–3] and high income [4, 5]), and region of residence.

For the subgroup analyses, we divided participants by age and sex (< 60 years old and ≥ 60 years old; males and females) and analyzed the crude model, model 1, and model 2. We additionally performed subgroup analyses according to cigarette smoking, alcohol drinking, obesity, blood pressure, total cholesterol, and fasting blood glucose using unconditional logistic regression.

Additionally, we evaluated these relationships according to hydrophilic or lipophilic statin use.

Two-tailed analyses were presented, and P values < 0.05 were set as the level of significance. SAS version 9.4 (SAS Institute Inc., Cary, NC, USA) was used for statistical analyses.

## Results

The days of previous statin use were greater in the thyroid cancer group than in the non-thyroid cancer group (72.3, standard deviation [SD] = 181.2 days vs. 64.3, SD = 174.4 days, P = 0.003, Table 1). Total cholesterol level, SBP, DBP, fasting blood glucose, obesity, smoking status, CCI scores, and dyslipidemia were different between the thyroid cancer group and the non-thyroid cancer group (P < 0.05).

**Table 1:** General characteristics of participants.

Characteristics	Total participants		P-value
	Thyroid cancer	Non-thyroid cancer	
**Age (years old, n, %)**			1.000
40–44	89 (1.6)	356 (1.6)	
45–49	771 (14.0)	3084 (14.0)	
50–54	1521 (27.7)	6084 (27.7)	
55–59	1295 (23.5)	5180 (23.5)	
60–64	862 (15.7)	3448 (15.7)	
65–69	547 (9.9)	2188 (9.9)	
70–74	304 (5.5)	1216 (5.5)	
75–79	87 (1.6)	348 (1.6)	
80–84	24 (0.4)	96 (0.4)	
85+	1 (0.0)	4 (0.0)	
**Sex (n, %)**			1.000
Males	1167 (21.2)	4668 (21.2)	
Females	4334 (78.8)	17,336 (78.8)	
**Income (n, %)**			1.000
1 (lowest)	693 (12.6)	2772 (12.6)	
2	632 (11.5)	2528 (11.5)	
3	852 (15.5)	3408 (15.5)	
4	1117 (20.3)	4468 (20.3)	
5 (highest)	2207 (40.1)	8828 (40.1)	
**Region of residence (n, %)**			1.000
Urban	2646 (48.1)	10,584 (48.1)	
Rural	2855 (51.9)	11,420 (51.9)	
Total cholesterol (mg/dL, mean, SD)	199.2 (37.7)	201.8 (38.0)	< 0.001^†^
SBP (mmHg, mean, SD)	124.4 (15.9)	123.6 (16.2)	0.001^†^
DBP (mmHg, mean, SD)	77.5 (10.5)	76.8 (10.5)	< 0.001^†^
Fasting blood glucose (mg/dL, mean, SD)	97.4 (23.0)	98.3 (26.3)	0.008^†^
**Obesity (n, %)**^**a**^			< 0.001*
Underweight	72 (1.3)	485 (2.2)	
Normal	1810 (32.9)	8270 (37.6)	
Overweight	1567 (28.5)	6002 (27.3)	
Obese I	1819 (33.1)	6523 (29.6)	
Obese II	233 (4.2)	724 (3.3)	
**Smoking status (n, %)**			< 0.001*
Nonsmoker	4811 (87.5)	18,871 (85.8)	
Past smoker	380 (6.9)	1300 (5.9)	
Current smoker	310 (5.6)	1833 (8.3)	
**Alcohol consumption (n, %)**			0.338
< 1 time a week	4283 (77.9)	16,999 (77.3)	
≥ 1 time a week	1218 (22.1)	5005 (22.8)	
**CCI score (score, n, %)**^**b**^			< 0.001*
0	3359 (61.1)	17,078 (77.6)	
1	890 (16.2)	2775 (12.6)	
2	325 (5.9)	1100 (5.0)	
3	119 (2.2)	474 (2.2)	
≥ 4	808 (14.7)	577 (2.6)	
Dyslipidemia (n, %)	1893 (34.4)	6119 (27.8)	< 0.001*
The dates of statin prescription (days, mean, SD)	72.3 (181.2)	64.3 (174.4)	0.003^†^

*CCI* Charlson comorbidity index, *DBP* diastolic blood pressure, *SBP* systolic blood pressure, *SD* standard deviation.

*Chi-square test. Significance at P < 0.05.

^†^Independent *t* test. Significance at P < 0.05.

^a^Obesity (BMI, body mass index, kg/m^2^) was categorized as < 18.5 (underweight), ≥ 18.5 to < 23 (normal), ≥ 23 to < 25 (overweight), ≥ 25 to < 30 (obese I), and ≥ 30 (obese II).

^b^CCI scores were calculated without thyroid cancer.

The thyroid cancer group showed higher odds for previous statin use in the crude model (OR = 1.10, 95% CI 1.04–1.17, P = 0.002, Table 2). However, the odds of previous statin use for thyroid cancer were low in model 1 (adjusted OR = 0.92, 95% CI 0.85–0.98, P = 0.014) and in model 2 (adjusted OR = 0.89, 95% CI 0.82–0.95, P = 0.001).

**Table 2:** Odds ratios (95% confidence interval) of the date of statin prescription (per 1 year) for thyroid cancer with subgroup analyses according to age, sex, income, and region of residence.

Characteristics	Odds ratios for thyroid cancer					
	Crude^a^	P-value	Model 1^a,b^	P-value	Model 2^a,c^	P-value
**Total participants (n = 27,505)**						
Statin prescription (per 1 year)	1.10 (1.04–1.17)	0.002*	0.92 (0.85–0.98)	0.014*	0.89 (0.82–0.95)	0.001*
**Age < 60 years old, males (n = 3905)**						
Statin prescription (per 1 year)	0.95 (0.78–1.16)	0.642	0.74 (0.59–0.93)	0.011*	0.70 (0.55–0.88)	0.003*
**Age < 60 years old, females (n = 14,475)**						
Statin prescription (per 1 year)	1.14 (1.02–1.26)	0.019*	0.92 (0.81–1.04)	0.163	0.88 (0.78–1.00)	0.057
**Age ≥ 60 years old, males (n = 1930)**						
Statin prescription (per 1 year)	1.17 (0.97–1.41)	0.113	1.03 (0.83–1.28)	0.803	0.96 (0.76–1.22)	0.760
**Age ≥ 60 years old, females (n = 7195)**						
Statin prescription (per 1 year)	1.10 (1.01–1.20)	0.038*	0.94 (0.84–1.04)	0.219	0.91 (0.81–1.01)	0.077
**Low income (n = 10,885)**						
Statin prescription (per 1 year)	1.11 (1.00–1.22)	0.048*	0.90 (0.80–1.01)	0.079	0.86 (0.77–0.98)	0.017*
**High income (n = 16,620)**						
Statin prescription (per 1 year)	1.10 (1.02–1.19)	0.016*	0.92 (0.84–1.01)	0.078	0.90 (0.82–0.98)	0.019*
**Urban (n = 13,230)**						
Statin prescription (per 1 year)	1.08 (0.98–1.17)	0.108	0.88 (0.80–0.97)	0.014*	0.86 (0.77–0.95)	0.004*
**Rural (n = 14,275)**						
Statin prescription (per 1 year)	1.13 (1.04–1.23)	0.005*	0.95 (0.86–1.05)	0.312	0.91 (0.82–1.01)	0.068

*CCI* Charlson comorbidity index, *DBP* diastolic blood pressure, *SBP* systolic blood pressure.

*Conditional logistic regression, significance at P < 0.05.

^a^Models were stratified by age, sex, income, and region of residence.

^b^Model 1 was adjusted for SBP, DBP, dyslipidemia history, total cholesterol and fasting blood glucose.

^c^Model 2 was adjusted for model 1 plus CCI scores, smoking, alcohol consumption, and obesity.

According to age and sex, the < 60-year-old male group showed 0.70 times the odds for thyroid cancer in the group with previous statin use in model 2 (95% CI 0.55–0.88, P = 0.003). The OR was 0.86 (95% CI 0.77–0.98, P = 0.017) for the low-income group and 0.90 (95% CI 0.82–0.98, P = 0.019) for the high-income group. According to region of residence, the urban group demonstrated low odds of thyroid cancer in the previous statin use group in model 2 (adjusted OR = 0.86, 95% CI 0.77–0.95, P = 0.004). The < 60 year old female, ≥ 60 year old male, ≥ 60 year old female, and rural subgroups did not show significant relationships between previous statin use and thyroid cancer.

**Figure 2:**
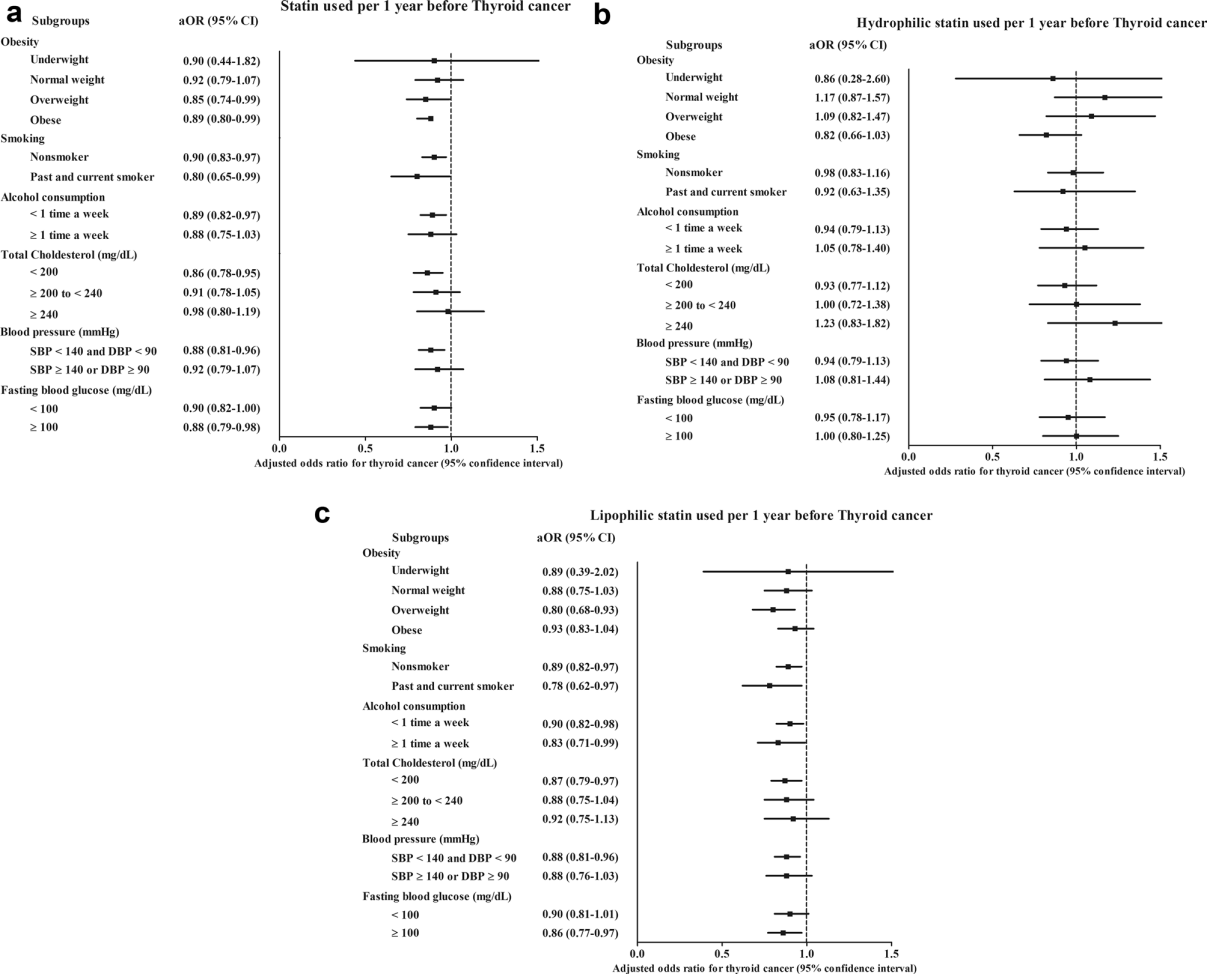
The odds ratios (95% confidence intervals) of (**a**) statin, (**b**) hydrophilic statin, and (**c**) lipophilic statin use for thyroid cancer according to obesity, smoking, alcohol consumption, total cholesterol, blood pressure, and fasting blood glucose.

Additional subgroup analyses demonstrated low odds of previous statin use associated with thyroid cancer in patients who were overweight, obese, nonsmokers, past smokers, and current smokers and who had lower alcohol consumption (< 1 times a week), < 200 mg/dL total cholesterol, normal blood pressure (SBP < 140 and DBP < 90), < 100 mg/dL fasting blood glucose, and ≥ 100 mg/dL fasting blood glucose (Fig. 2a and Table S1). The underweight, normal weight, higher alcohol consumption (≥ 1 time per week), normal total cholesterol level, and abnormal blood pressure level subgroups did not show a significant association between previous statin use and thyroid cancer (Table S1).

Statins were divided into hydrophilic and lipophilic groups, and their association with thyroid cancer was evaluated (Tables 3 and 4). Hydrophilic statins were not associated with thyroid cancer (Table 3). All subgroup analyses demonstrated identical results regarding the relationship of hydrophilic statins with thyroid cancer (Table 3, Fig. 2b, and Table S2). However, lipophilic statins were associated with decreased odds of thyroid cancer (adjusted OR = 0.88, 95% CI 0.81–0.95, P = 0.001, Table 4). Subgroups consisting of all ages and sexes and including subjects who had low income, had urban or rural residence, were overweight, were nonsmokers or past smokers/current smokers, consumed alcohol < 1 time per week or ≥ 1 time per week, and had total cholesterol < 200 mg/dL, normal blood pressure, and high fasting blood glucose showed negative associations between lipophilic statin use and thyroid cancer (Table 4, Fig. 2c, and Table S3).

**Table 3:** Odds ratios (95% confidence interval) of the date of hydrophilic statin prescription (per 1 year) for thyroid cancer with subgroup analyses according to age, sex, income, and region of residence.

Characteristics	Odds ratios for thyroid cancer					
	Crude^a^	P-value	Model 1^a,b^	P-value	Model 2^a,c^	P-value
**Total participants (n = 26,495)**						
Statin prescription (per 1 year)	1.15 (0.99–1.32)	0.060	0.99 (0.85–1.14)	0.843	0.97 (0.83–1.13)	0.689
**Age < 60 years old, males (n = 3805)**						
Statin prescription (per 1 year)	0.97 (0.76–1.25)	0.815	0.80 (0.62–1.03)	0.088	0.78 (0.60–1.02)	0.069
**Age < 60 years old, females (n = 13,935)**						
Statin prescription (per 1 year)	1.26 (1.06–1.50)	0.011*	1.11 (0.93–1.33)	0.255	1.09 (0.91–1.31)	0.352
**Age ≥ 60 years old, males (n = 1870)**						
Statin prescription (per 1 year)	1.10 (0.80–1.50)	0.567	0.93 (0.67–1.28)	0.648	0.80 (0.57–1.12)	0.194
**Age ≥ 60 years old, females (n = 6885)**						
Statin prescription (per 1 year)	1.16 (0.99–1.37)	0.068	1.00 (0.85–1.18)	0.993	1.01 (0.86–1.20)	0.877
**Low income (n = 10,475)**						
Statin prescription (per 1 year)	1.38 (1.09–1.75)	0.007*	1.18 (0.93–1.50)	0.180	1.14 (0.89–1.47)	0.301
**High income (n = 16,020)**						
Statin prescription (per 1 year)	1.04 (0.87–1.25)	0.682	0.90 (0.74–1.08)	0.249	0.89 (0.74–1.08)	0.241
**Urban (n = 12,730)**						
Statin prescription (per 1 year)	1.08 (0.88–1.32)	0.483	0.91 (0.74–1.13)	0.388	0.90 (0.72–1.12)	0.327
**Rural (n = 13,765)**						
Statin prescription (per 1 year)	1.22 (1.00–1.49)	0.049*	1.06 (0.87–1.31)	0.552	1.04 (0.84–1.28)	0.714

*CCI* Charlson comorbidity index, *DBP* diastolic blood pressure, *SBP* systolic blood pressure.

*Conditional logistic regression, Significance at P < 0.05.

^a^Models were stratified by age, sex, income, and region of residence.

^b^A model 1 was adjusted for dyslipidemia history, total cholesterol, SBP, DBP, and fasting blood glucose.

^c^A model 2 was adjusted for model 1 plus obesity, smoking, alcohol consumption, and CCI scores.

**Table 4:** Odds ratios (95% confidence interval) of the date of lipophilic statin prescription (per 1 year) for thyroid cancer with subgroup analyses according to age, sex, income, and region of residence.

Characteristics	Odds ratios for thyroid cancer					
	Crude^a^	P-value	Model 1^a,b^	P-value	Model 2^a,c^	P-value
**Total participants (n = 26,495)**						
Statin prescription (per 1 year)	1.09 (1.02–1.17)	0.011*	0.91 (0.84–0.98)	0.013*	0.88 (0.81–0.95)	0.001*
**Age < 60 years old, males (n = 3805)**						
Statin prescription (per 1 year)	1.12 (1.01–1.23)	0.035*	0.90 (0.81–1.01)	0.077	0.87 (0.77–0.97)	0.017*
**Age < 60 years old, females (n = 13,935)**						
Statin prescription (per 1 year)	1.07 (0.98–1.17)	0.119	0.91 (0.83–1.01)	0.083	0.89 (0.80–0.98)	0.023*
**Age ≥ 60 years old, males (n = 1870)**						
Statin prescription (per 1 year)	1.05 (0.90–1.22)	0.554	0.87 (0.73–1.03)	0.102	0.84 (0.70–1.00)	0.049*
**Age ≥ 60 years old, females (n = 6885)**						
Statin prescription (per 1 year)	1.10 (1.02–1.19)	0.010*	0.92 (0.85–1.00)	0.053	0.89 (0.81–0.97)	0.007*
**Low income (n = 10,475)**						
Statin prescription (per 1 year)	1.06 (0.95–1.18)	0.310	0.85 (0.75–0.97)	0.014*	0.82 (0.72–0.94)	0.003*
**High income (n = 16,020)**						
Statin prescription (per 1 year)	1.11 (1.02–1.21)	0.014*	0.94 (0.86–1.03)	0.207	0.91 (0.83–1.00)	0.060
**Urban (n = 12,730)**						
Statin prescription (per 1 year)	1.07 (0.98–1.18)	0.151	0.89 (0.80–0.99)	0.032*	0.87 (0.78–0.97)	0.012*
**Rural (n = 13,765)**						
Statin prescription (per 1 year)	1.11 (1.01–1.22)	0.030*	0.93 (0.84–1.03)	0.175	0.89 (0.80–0.99)	0.035*

CCI, Charlson comorbidity index; DBP, diastolic blood pressure; SBP, systolic blood pressure.

*Conditional logistic regression, Significance at P < 0.05.

^a^Models were stratified by age, sex, income, and region of residence.

^b^A model 1 was adjusted for dyslipidemia history, total cholesterol, SBP, DBP, and fasting blood glucose.

^c^A model 2 was adjusted for model 1 plus obesity, smoking, alcohol consumption, and CCI scores.

## Discussion

Previous statin use was associated with a lower rate of thyroid cancer in the ≥ 40-year-old population. Although the mean days of previous statin use was higher in the thyroid cancer group than in the non-thyroid cancer group, previous statin use was negatively related to thyroid cancer after adjusting for past medical history, including histories of hyperlipidemia, obesity, smoking, and alcohol consumption. The negative association of previous statin use with thyroid cancer was consistent in the < 60-year-old male population and other subgroups by covariates. To our knowledge, this is the largest cohort study to examine the association of statin use with thyroid cancer.

In line with the current results, a few previous experimental studies have supported the anticancer effects of statins in thyroid cancer. Lovastatin treatment suppressed proliferation and promoted apoptosis in a thyroid cell line^17^. The authors suggested that statins, as HMG-CoA reductases, inhibited the prenylation of proteins, such as p21-RAS^17^. Moreover, lovastatin treatment in combination with troglitazone exhibited antiproliferation effects in a mouse xenografted anaplastic thyroid cancer model, which might be due to the effects of cell cycle arrest at the G0/G1 phases and reduction in hyperphosphorylated retinoblastoma protein-E2F1 signaling^18^. In a retrospective study, statin treatment for 5 or more years was related to a lower rate of thyroid nodules in dyslipidemic patients (adjusted OR = 0.312, 95% CI 0.156–0.625, P < 0.001)^28^. In addition to the direct effects of statins on thyroid cancer cells, the indirect effects of statins on lipid-lowering and anti-inflammation could be beneficial for reducing thyroid cancer. Obesity has been reported to increase the risk of thyroid cancer in preclinical and clinical studies^29,30^. In a mouse model study, obesity mediated thyroid carcinogenesis by increasing insulin resistance, oxidative stress, leptin, and cytokines related to inflammation^29^. A review study showed a consistent association of obesity with a high rate of thyroid cancer in multiple epidemiologic studies^30^. Statin use could decrease the risk of thyroid cancer by attenuating obesity-related metabolic problems.

On the other hand, a previous case–control study reported increased odds of thyroid cancer development in individuals with prior statin use (adjusted OR = 1.39, 95% CI 1.08–1.78)^21^, which was similar to our results in the crude model. The longer duration of previous statin use in the thyroid cancer group in the crude model may originate from detection bias. Because the participants with statin use might visit clinics more frequently, statin users could have a greater chance of having their thyroid cancer detected. This study investigated a longer period of statin use (2 years) than a previous study (6 months)^21^. Because the effects of statin use can last for approximately 2 years^31^, a longer period of statin use needs to be assessed. Thus, the possible effects of detection bias were higher than those in a previous study. Nevertheless, previous statin use showed a negative association with thyroid cancer after adjusting for comorbidities, obesity, smoking, and alcohol consumption. However, the previous study did not consider these possible confounders.

According to age and sex, only the < 60-year-old male group demonstrated the negative association of previous statin use with thyroid cancer in the present study. There was no statistically significant protective anticancer effect of statins in the subgroups of women. A previous study even showed increased odds of previous statin use for thyroid cancer in women^21^. This sex-specific effect of statins on thyroid cancer could be explained by the impacts of estrogen on both thyroid cancer and statin effects. Females have a higher incidence rate of thyroid cancer, which has been partially attributed to the interaction of estrogen with thyroid cancer. Estrogen was suggested as a potent mitogen for thyroid tumor cells^13^. Estrogen promoted the proliferation of thyroid tumor cells and activated the mitogen-activated protein (MAP) kinase pathway by increasing the expression of cyclin D1 protein^13^. In addition, estrogen has multiple effects on the tumor microenvironment of thyroid cancer, including inflammation, hypoxia, angiogenesis, and metastasis^32^. In addition to its effects on thyroid cancer, estrogen was reported to have inhibitory effects on the cell cycle arrest caused by statins^13^. Moreover, estrogen is involved in metabolic pathways identical to those of statins, including cytochrome enzymes and transporters, so it can competitively inhibit the effects of statins^33^. In addition to a younger age among males, overweight, obese, nonsmoker, past smoker, current smoker, low alcohol consumption, normal total cholesterol, normal blood pressure, normal fasting blood glucose, and high fasting blood glucose showed a negative association with previous statin use and thyroid cancer in the present study, which implied the solid relationship of previous statin use with the decreased rate of thyroid cancer. Some subgroups including participants with underweight status, normal weight, higher alcohol consumption (≥ 1 time per week), normal total cholesterol levels, and abnormal blood pressure levels did not show a statistically significant association with previous statin use in the present study. The relatively small subgroup size could attenuate the statistical power to detect a relationship between statin use and thyroid cancer. In the analysis according to statin type, lipophilic statins, but not hydrophilic statins, were associated with decreased odds of thyroid cancer in the present study. Previous studies suggested that lipophilic statins were more effective in preventing cancer progression because they could be dissolved in the cellular membrane and diffuse to extrahepatic tissues^34^. Thus, it can be presumed that the high bioavailability of lipophilic statins allowed them to more effectively reduce the likelihood of thyroid cancer than hydrophilic statins.

In the large study population, possible confounders such as comorbidities and the life-habit factors of cigarette smoking, alcohol drinking, and BMI were adjusted in the current study. Subgroup analyses were extensively conducted for factors that could be related to thyroid cancer and/or statin use, including age, sex, income, region of residence, obesity, smoking, alcohol consumption, total cholesterol, blood pressure, and fasting blood glucose. However, largely due to the use of health insurance data, a number of limitations need to be considered when interpreting the present results. The types and stages of thyroid cancer were heterogeneous in this study population. Information on the TNM stages of thyroid cancer was not available in the National Health Insurance Service data. Because thyroid cancer progresses slowly, latent thyroid cancer could be present before the index date of the present study. However, thyroid cancer is identified in Korea early because of massive thyroid cancer screening^35^. Thus, the effects of latent thyroid cancer before the index date might not have a substantial effect in this study. Because previous statin use was based on prescription data, the compliance of drug use could not be determined. Although the previous use of statins was assessed over a period of 2 years, the initial date of statin use could not be determined in the present study. Thus, the duration of statin use could have some variations among participants. In addition, this study did not assess the adverse effects of statin use. A previous study reported dose-dependent unintended adverse effects of statins, such as liver dysfunction and renal failure^36^. Future studies are warranted to delineate the current limitations.

In conclusion, previous statin use was related to a decreased rate of thyroid cancer when adjusted for comorbidities in ≥ 40-year-old Koreans. This relationship between statin use and thyroid cancer was consistent in many subgroups and for lipophilic statin use but not in the female population or for hydrophilic statin use.

## Supplementary Information

Supplementary Tables.
