# Supplementary material for: Evaluation of the relationship between previous statin use and thyroid cancer using Korean National Health Insurance Service-Health Screening Cohort data
Source: Sci Rep. 2021 Apr 12;11:7912. doi: 10.1038/s41598-021-87297-6 (PMC8041859; doi:10.1038/s41598-021-87297-6)
Supplement: Supplementary file 1 — Supplementary Tables. [file 41598_2021_87297_MOESM1_ESM.docx]

**Evaluation of the relationship between previous statin use and thyroid cancer using Korean National Health Insurance Service-Health Screening Cohort data**

So Young Kim, MD, PhD^1^, Young Shin Song, MD, PhD^2^, Jee Hye Wee, MD, PhD^3^, Chanyang Min, PhD^4,5^, Dae Myoung Yoo, MS^4^, Chang-Ho Lee, MD, PhD^1^, Chang Myeon Song, MD, PhD^6^, Bumjung Park MD, PhD^3^, Hyo Geun Choi, MD, PhD^3,4*^

^1^Department of Otorhinolaryngology-Head & Neck Surgery, CHA Bundang Medical Center, CHA University, Seongnam, Korea

^2^Department of Internal Medicine, CHA Bundang Medical Center, CHA University, Seongnam, Korea

^3^Department of Otorhinolaryngology-Head & Neck Surgery, Hallym University College of Medicine, Anyang, Korea

^4^Hallym Data Science Laboratory, Hallym University College of Medicine, Anyang, Korea

^5^Graduate School of Public Health, Seoul National University, Seoul, Korea

^6^Department of Otorhinolaryngology-Head & Neck Surgery, Hanyang University College of Medicine, Seoul, Korea

**Running title:** Statin and thyroid cancer

***Correspondence:** pupen@naver.com

**S1 Table** Odds ratios (95% confidence interval) of the date of statin prescription (per 1 year) for thyroid cancer in each subgroup according to obesity, smoking, alcohol consumption, total cholesterol, blood pressure, and fasting blood glucose

| Characteristics | | | Odds ratios of statins prescription (per 1 year) for thyroid cancer | | | | | | | | | | | |
| --- | --- | --- | --- | --- | --- | --- | --- | --- | --- | --- | --- | --- | --- | --- |
|  |  |  | | Model 1† | | P-value | | Model 2‡ | | P-value | | Model 3§ | | P-value |
| Obesity | | | | | | | | | | | | | | |
|  | Underweight (n = 557) | | 1.44 (0.80-2.62) | | 0.228 | | 0.81 (0.41-1.62) | | 0.551 | | 0.86 (0.43-1.74) | | 0.683 | |
|  | Normal weight (n = 10,080) | | 1.09 (0.96-1.24) | | 0.175 | | 0.92 (0.79-1.06) | | 0.233 | | 0.93 (0.80-1.07) | | 0.309 | |
|  | Overweight (n = 7,569) | | 1.04 (0.92-1.17) | | 0.531 | | 0.88 (0.76-1.01) | | 0.066 | | 0.84 (0.73-0.97) | | 0.017* | |
|  | Obese (n = 9,299) | | 1.08 (0.99-1.18) | | 0.089 | | 0.91 (0.82-1.00) | | 0.060 | | 0.89 (0.80-0.99) | | 0.032* | |
| Smoking | | |  | |  | |  | |  | |  | |  | |
|  | Nonsmoker (n = 23,682) | | 1.10 (1.03-1.18) | | 0.003* | | 0.92 (0.86-1.00) | | 0.042* | | 0.90 (0.83-0.97) | | 0.008* | |
|  | Past smoker and current smoker (n = 3,823) | | 1.11 (0.94-1.31) | | 0.237 | | 0.86 (0.71-1.04) | | 0.127 | | 0.79 (0.64-0.97) | | 0.022* | |
| Alcohol consumption | | |  | |  | |  | |  | |  | |  | |
|  | < 1 time a week (n = 21,282) | | 1.12 (1.04-1.20) | | 0.002* | | 0.93 (0.86-1.01) | | 0.068 | | 0.89 (0.82-0.97) | | 0.008* | |
|  | ≥ 1 time a week (n = 6,223) | | 1.06 (0.93-1.20) | | 0.384 | | 0.87 (0.75-1.00) | | 0.056 | | 0.86 (0.74-1.01) | | 0.061 | |
| Total cholesterol (mg/dL) | | |  | |  | |  | |  | |  | |  | |
|  | < 200 (n = 14,032) | | 1.08 (1.00-1.17) | | 0.049* | | 0.89 (0.81-0.97) | | 0.012* | | 0.87 (0.79-0.95) | | 0.003* | |
|  | ≥ 200 to < 240 (n = 9,456) | | 1.11 (0.98-1.27) | | 0.110 | | 0.94 (0.81-1.08) | | 0.368 | | 0.90 (0.77-1.04) | | 0.163 | |
|  | ≥ 240 (n = 4,017) | | 1.19 (1.00-1.40) | | 0.046* | | 1.00 (0.83-1.21) | | 0.988 | | 0.97 (0.80-1.18) | | 0.781 | |
| Blood pressure (mmHg) | | |  | |  | |  | |  | |  | |  | |
|  | SBP < 140 and DBP < 90 (n = 22,144) | | 1.10 (1.02-1.18) | | 0.012* | | 0.91 (0.83-0.98) | | 0.018* | | 0.88 (0.81-0.96) | | 0.004* | |
|  | SBP ≥ 140 or DBP ≥ 90 (n = 5,361) | | 1.12 (0.99-1.26) | | 0.074 | | 0.94 (0.82-1.09) | | 0.419 | | 0.91 (0.79-1.05) | | 0.214 | |
| Fasting blood glucose (mg/dL) | | |  | |  | |  | |  | |  | |  | |
|  | < 100 (n = 18,714) | | 1.17 (1.08-1.27) | | <0.001* | | 0.94 (0.85-1.03) | | 0.174 | | 0.91 (0.82-1.00) | | 0.049* | |
|  | ≥ 100 (n = 8,791) | | 1.05 (0.95-1.14) | | 0.346 | | 0.90 (0.80-1.00) | | 0.040* | | 0.87 (0.78-0.97) | | 0.015* | |

Abbreviations: CCI, Charlson comorbidity index; DBP, diastolic blood pressure; SBP, systolic blood pressure

* Logistic regression, Significance at P < 0.05

† A model 1 was adjusted for age, sex, income, and region of residence.

‡ A model 2 was adjusted model 1 plus dyslipidemia history, total cholesterol, SBP, DBP, and fasting blood glucose.

§ A model 3 was adjusted for model 2 plus, obesity, smoking, alcohol consumption, and CCI scores.

**S2 Table** Odds ratios (95% confidence interval) of the date of hydrophilic statin prescription (per 1 year) for thyroid cancer in each subgroup according to obesity, smoking, alcohol consumption, total cholesterol, blood pressure, and fasting blood glucose

| Characteristics | | | Odds ratios of statins prescription (per 1 year) for thyroid cancer | | | | | | | | | | | |
| --- | --- | --- | --- | --- | --- | --- | --- | --- | --- | --- | --- | --- | --- | --- |
|  |  |  | | Model 1† | | P-value | | Model 2‡ | | P-value | | Model 3§ | | P-value |
| Obesity | | | | | | | | | | | | | | |
|  | Underweight (n = 534) | | 1.39 (0.50-3.84) | | 0.531 | | 0.86 (0.29-2.53) | | 0.779 | | 0.86 (0.28-2.60) | | 0.787 | |
|  | Normal weight (n = 9,718) | | 1.29 (0.96-1.71) | | 0.088 | | 1.11 (0.82-1.49) | | 0.507 | | 1.17 (0.87-1.57) | | 0.314 | |
|  | Overweight (n = 7,318) | | 1.23 (0.93-1.62) | | 0.148 | | 1.10 (0.83-1.47) | | 0.496 | | 1.09 (0.82-1.47) | | 0.549 | |
|  | Obese (n = 8,925) | | 0.99 (0.81-1.23) | | 0.949 | | 0.85 (0.69-1.06) | | 0.140 | | 0.82 (0.66-1.03) | | 0.086 | |
| Smoking | | |  | |  | |  | |  | |  | |  | |
|  | Nonsmoker (n = 22,772) | | 1.13 (0.96-1.32) | | 0.140 | | 0.97 (0.83-1.14) | | 0.733 | | 0.98 (0.83-1.16) | | 0.840 | |
|  | Past smoker and current smoker (n = 3,723) | | 1.31 (0.93-1.84) | | 0.121 | | 1.08 (0.76-1.53) | | 0.686 | | 0.92 (0.63-1.35) | | 0.680 | |
| Alcohol consumption | | |  | |  | |  | |  | |  | |  | |
|  | < 1 time a week (n = 20,427) | | 1.12 (0.94-1.32) | | 0.203 | | 0.96 (0.81-1.14) | | 0.629 | | 0.94 (0.79-1.13) | | 0.515 | |
|  | ≥ 1 time a week (n = 6,068) | | 1.23 (0.94-1.62) | | 0.128 | | 1.05 (0.79-1.39) | | 0.731 | | 1.05 (0.78-1.40) | | 0.757 | |
| Total cholesterol (mg/dL) | | |  | |  | |  | |  | |  | |  | |
|  | < 200 (n = 13,518) | | 1.09 (0.91-1.31) | | 0.340 | | 0.94 (0.78-1.13) | | 0.516 | | 0.93 (0.77-1.12) | | 0.424 | |
|  | ≥ 200 to < 240 (n = 9,105) | | 1.21 (0.89-1.64) | | 0.227 | | 1.04 (0.76-1.42) | | 0.816 | | 1.00 (0.72-1.38) | | 0.993 | |
|  | ≥ 240 (n = 3,872) | | 1.33 (0.91-1.94) | | 0.139 | | 1.17 (0.80-1.72) | | 0.419 | | 1.23 (0.83-1.82) | | 0.306 | |
| Blood pressure (mmHg) | | |  | |  | |  | |  | |  | |  | |
|  | SBP < 140 and DBP < 90 (n = 21,378) | | 1.12 (0.95-1.32) | | 0.188 | | 0.96 (0.81-1.14) | | 0.658 | | 0.94 (0.79-1.13) | | 0.508 | |
|  | SBP ≥ 140 or DBP ≥ 90 (n = 5,117) | | 1.23 (0.94-1.62) | | 0.134 | | 1.07 (0.81-1.42) | | 0.629 | | 1.08 (0.81-1.44) | | 0.593 | |
| Fasting blood glucose (mg/dL) | | |  | |  | |  | |  | |  | |  | |
|  | < 100 (n = 18,026) | | 1.21 (1.00-1.47) | | 0.056 | | 1.00 (0.82-1.22) | | 0.976 | | 0.95 (0.78-1.17) | | 0.654 | |
|  | ≥ 100 (n = 8,469) | | 1.09 (0.88-1.35) | | 0.418 | | 0.97 (0.78-1.21) | | 0.793 | | 1.00 (0.80-1.25) | | 0.968 | |

Abbreviations: CCI, Charlson comorbidity index; DBP, diastolic blood pressure; SBP, systolic blood pressure

* Logistic regression, Significance at P < 0.05

† A model 1 was adjusted for age, sex, income, and region of residence.

‡ A model 2 was adjusted model 1 plus dyslipidemia history, total cholesterol, SBP, DBP, and fasting blood glucose.

§ A model 3 was adjusted for model 2 plus, obesity, smoking, alcohol consumption, and CCI scores.

**S3 Table** Odds ratios (95% confidence interval) of the date of lipophilic statin prescription (per 1 year) for thyroid cancer in each subgroup according to obesity, smoking, alcohol consumption, total cholesterol, blood pressure, and fasting blood glucose

| Characteristics | | | Odds ratios of statins prescription (per 1 year) for thyroid cancer | | | | | | | | | | | |
| --- | --- | --- | --- | --- | --- | --- | --- | --- | --- | --- | --- | --- | --- | --- |
|  |  |  | | Model 1† | | P-value | | Model 2‡ | | P-value | | Model 3§ | | P-value |
| Obesity | | | | | | | | | | | | | | |
|  | Underweight (n = 534) | | 1.46 (0.71-2.98) | | 0.305 | | 0.82 (0.37-1.84) | | 0.628 | | 0.89 (0.39-2.02) | | 0.785 | |
|  | Normal weight (n = 9,718) | | 1.05 (0.92-1.21) | | 0.463 | | 0.88 (0.76-1.03) | | 0.112 | | 0.88 (0.75-1.03) | | 0.116 | |
|  | Overweight (n = 7,318) | | 1.00 (0.88-1.15) | | 0.965 | | 0.84 (0.72-0.98) | | 0.023* | | 0.80 (0.68-0.93) | | 0.005* | |
|  | Obese (n = 8,925) | | 1.10 (1.00-1.21) | | 0.056 | | 0.94 (0.84-1.04) | | 0.240 | | 0.93 (0.83-1.04) | | 0.183 | |
| Smoking | | |  | |  | |  | |  | |  | |  | |
|  | Nonsmoker (n = 22,772) | | 1.10 (1.02-1.18) | | 0.010* | | 0.92 (0.85-1.00) | | 0.050 | | 0.89 (0.82-0.97) | | 0.008* | |
|  | Past smoker and current smoker (n = 3,723) | | 1.05 (0.87-1.28) | | 0.589 | | 0.82 (0.66-1.01) | | 0.063 | | 0.78 (0.62-0.97) | | 0.027* | |
| Alcohol consumption | | |  | |  | |  | |  | |  | |  | |
|  | < 1 time a week (n = 20,427) | | 1.11 (1.03-1.20) | | 0.005* | | 0.93 (0.85-1.01) | | 0.093 | | 0.90 (0.82-0.98) | | 0.013* | |
|  | ≥ 1 time a week (n = 6,068) | | 1.02 (0.88-1.17) | | 0.806 | | 0.83 (0.71-0.98) | | 0.028* | | 0.83 (0.71-0.99) | | 0.032* | |
| Total cholesterol (mg/dL) | | |  | |  | |  | |  | |  | |  | |
|  | < 200 (n = 13,518) | | 1.08 (0.99-1.17) | | 0.084 | | 0.89 (0.81-0.99) | | 0.023* | | 0.87 (0.79-0.97) | | 0.008* | |
|  | ≥ 200 to < 240 (n = 9,105) | | 1.10 (0.95-1.27) | | 0.218 | | 0.92 (0.78-1.08) | | 0.282 | | 0.88 (0.75-1.04) | | 0.134 | |
|  | ≥ 240 (n = 3,872) | | 1.15 (0.96-1.37) | | 0.141 | | 0.96 (0.78-1.17) | | 0.674 | | 0.92 (0.75-1.13) | | 0.429 | |
| Blood pressure (mmHg) | | |  | |  | |  | |  | |  | |  | |
|  | SBP < 140 and DBP < 90 (n = 21,378) | | 1.09 (1.01-1.18) | | 0.030* | | 0.90 (0.83-0.99) | | 0.023* | | 0.88 (0.81-0.96) | | 0.006* | |
|  | SBP ≥ 140 or DBP ≥ 90 (n = 5,117) | | 1.09 (0.96-1.24) | | 0.202 | | 0.92 (0.80-1.07) | | 0.283 | | 0.88 (0.76-1.03) | | 0.117 | |
| Fasting blood glucose (mg/dL) | | |  | |  | |  | |  | |  | |  | |
|  | < 100 (n = 18,026) | | 1.16 (1.06-1.27) | | 0.001* | | 0.93 (0.84-1.03) | | 0.144 | | 0.90 (0.81-1.01) | | 0.061 | |
|  | ≥ 100 (n = 8,469) | | 1.03 (0.94-1.14) | | 0.501 | | 0.89 (0.80-1.00) | | 0.047* | | 0.86 (0.77-0.97) | | 0.012* | |

Abbreviations: CCI, Charlson comorbidity index; DBP, diastolic blood pressure; SBP, systolic blood pressure

* Logistic regression, Significance at P < 0.05

† A model 1 was adjusted for age, sex, income, and region of residence.

‡ A model 2 was adjusted model 1 plus dyslipidemia history, total cholesterol, SBP, DBP, and fasting blood glucose.

§ A model 3 was adjusted for model 2 plus, obesity, smoking, alcohol consumption, and CCI scores.
